# Editorial: Advances in *Aspergillus fumigatus* Pathobiology

**DOI:** 10.3389/fmicb.2016.00043

**Published:** 2016-02-03

**Authors:** Frédéric Lamoth, Praveen R. Juvvadi, William J. Steinbach

**Affiliations:** ^1^Division of Pediatric Infectious Diseases, Department of Pediatrics, Duke University Medical CenterDurham, NC, USA; ^2^Infectious Diseases Service, Department of Medicine, Lausanne University HospitalLausanne, Switzerland; ^3^Institute of Microbiology, Lausanne University HospitalLausanne, Switzerland; ^4^Department of Molecular Genetics and Microbiology, Duke University Medical CenterDurham, NC, USA

**Keywords:** invasive aspergillosis, antifungal resistance, *Aspergillus fumigatus*, fungal virulence, fungal proteins

*Aspergillus fumigatus* and other *Aspergillus* spp. are ubiquitous in our environment. However, their potential to cause severe disease in humans was ignored for many centuries. The first reported case of presumed human aspergillosis is from 1791 during the French revolution. A 22-year old soldier sought medical attention for painful cheek swelling because of a fungus ball of the maxillary sinus invading the mouth and orbit (Plaignaud, 1791). The patient ultimately recovered after surgery. A few decades earlier, in 1729, the Italian priest and botanist Pier Antonio Micheli provided the first description of the fungal genus that he named “*Aspergillus*” because of the similarity of the conidial head and spores to the aspergillum, the liturgical implement used to sprinkle holy water in the Catholic church (Micheli, 1729). During the first half of the twentieth century, *Aspergillus* spp. were considered common laboratory contaminants and only an occasional cause of human diseases with some case reports of chronic bronchopulmonary aspergillosis among farmers, cerebral abscesses, meningitis, and bone infections (Cawley, 1947). The introduction of steroid therapy in the 1950s, the later development of anti-neoplastic chemotherapy, and the first hematopoietic stem cell transplantations during the following decades revealed the devastating potential of these fungi in patients with severely depressed immune systems.

Invasive aspergillosis (IA) has now emerged as a major infectious threat and the prevalence and spectrum of the disease has progressed in parallel with advances in medicine and the advent of new therapies with potent immunosuppressive effects. The cumulative 12-month incidence of IA is estimated at 1.6% in hematopoietic stem cell transplant recipients and 0.7% in solid organ transplant recipients, with an overall 1-year mortality of 40–75% (Kontoyiannis et al., 2010; Pappas et al., 2010). Moreover, IA is increasingly reported in populations with other underlying conditions, such as intensive-care unit patients, or patients with autoimmune or chronic bronchopulmonary diseases (Meersseman et al., 2004; Garbino et al., 2011).

Despite a slight improvement in survival rates (Steinbach et al., 2012), the mortality of IA remains high, and little significant progress has been made in the management of the disease over the last several decades. Amphotericin B was historically the pillar of antifungal therapy, but included an unacceptably high rate of failure due to toxicity. At the beginning of this century, voriconazole demonstrated a better efficacy and safety profile and became the preferred first-line therapy of IA (Herbrecht et al., 2002). However, emergence of resistance to triazoles as a probable consequence of the widespread use of fungicides in the agriculture and industry (Vermeulen et al., 2013) has led to the need for second-line antifungal agents. Echinocandins, such as caspofungin or micafungin, are now considered as salvage therapy of IA (Maertens et al., 2004), but their lack of fungicidal activity limits their efficacy. Posaconazole is active against most *Aspergillus* spp. However, the most frequent mechanism of voriconazole resistance (i.e., mutations of the *Cyp51A* gene) often confers pan-azole resistance and the increasing use of posaconazole prophylaxis in patients with hematologic malignancies raises concern about breakthrough infections due to resistant *Aspergillus* spp. or other fungi (Auberger et al., 2012). Combination antifungal therapy, such as the association of triazoles and echinocandins, has led to inconclusive results (Marr et al., 2015).

Considerable effort from the research community is dedicated to the discovery of new antifungal targets. An effective antifungal agent must be fungal-specific to avoid unacceptable human toxicity, but this is difficult to achieve as both fungi and humans are eukaryotes. Indeed, all currently approved antifungal drugs target the specific components of the fungal cell membrane (ergosterol) or cell wall (β-1,3-D-glucan). A better understanding of the molecular pathways involved in fungal metabolism, virulence, stress response, and resistance are therefore important steps toward the discovery of novel therapeutic approaches.

The purpose of this research topic is to provide an overview on the current state of research and to strengthen the links within the *Aspergillus* community. Scientists purposefully chosen from various countries and continents have contributed to this special issue dealing with all the medical aspects of IA, including taxonomy, genetics, epidemiology, pathogenesis, antifungal resistance, and novel therapeutic perspectives. Multiple species have now been identified within the *Aspergillus* section *Fumigati* and the possible relationship between their metabolite profiles and pathogenicity are discussed (Frisvad and Larsen). The worldwide problem of emerging azole resistance among *Aspergillus* spp. is illustrated by an interesting epidemiological study and update of the situation in Asia (Chowdhary et al.). In addition, the existence of mechanisms of azole resistance other than mutations of the *Cyp51A* gene, such as increased expression of ATP-binding cassette (ABC) transporters, is highlighted (Moye-Rowley). To counteract emerging resistance, novel potential antifungal targets are being investigated and several research groups present their latest updates on the cell wall integrity signaling pathway (Valiante et al.), the Ras pathway (Al Abdallah and Fortwendel), the Hsp90-calcineurin network (Juvvadi et al.; Lamoth et al.), and the regulation of zinc and iron homeostasis (Schafferer et al.; Vicentefranqueira et al.). Finally, because innate and adaptive immunity are key determinants in the development of IA, the analysis of host-pathogen interactions represents a promising research area. The current knowledge about the immune responses mediated via T-helper cells is presented in two reviews (Amarsaikhan and Templeton; Thakur et al.). The recent identification of host genetic determinants of IA, such as TLRs polymorphisms, also opens perspectives for preventive strategies (Oliveira-Coelho et al.).

This overview on the recent advances in *A. fumigatus* pathobiology suggests that we are entering a new era in the approach and management of IA. The epidemiology and pathophysiology of the disease has become more complex, with emerging resistance to triazoles and the increased diversity of immunosuppression types and host susceptibilities. Combination therapies of existing compounds or novel molecules that may enhance their activity or modulate the pattern of host immune recognition, as well as personalized diagnostic and therapeutic strategies based on individual susceptibility profiles of high risk patients, may change our conventional approach of IA and hopefully result in better outcomes.

## Author contributions

FL: Design and writing; PJ: Design and writing; WS: Design and writing.

### Conflict of interest statement

The authors declare that the research was conducted in the absence of any commercial or financial relationships that could be construed as a potential conflict of interest.
